# A Comprehensive Understanding of Knife Cutting: Effects of Hardness, Blade Angle and the Micro-Geometry of Blade Edge on the Cutting Performance

**DOI:** 10.3390/ma16155375

**Published:** 2023-07-31

**Authors:** Qinyi Zhang, Feng Liu, Dong Wu, Shikang Qu, Wei Liu, Zhangxiao Chen

**Affiliations:** 1 School of Materials Science and Engineering, Wuhan University of Technology, Wuhan 430070, China; zhqy@whut.edu.cn (Q.Z.); liufeng327xt@163.com (F.L.); 15271417642@163.com (S.Q.); 2 Yangjiang Advanced Alloys Laboratory, Yangjiang 529500, China; 3 Yangjiang Tuobituo Industrial Technology Research Institute Co., Ltd., Yangjiang 529500, China; lw@tuobituo.com (W.L.); czx@tuobituo.com (Z.C.)

**Keywords:** edge micro-geometry, sharpness retention, knife blade, carbides, martensitic steels

## Abstract

The cutting performance of steel blades is an eternal, attractive topic in the knife industry. It is a complicated process to cut up materials because it usually involves the contact mechanics of the material been cut, the geometry and roughness of the blade edge and the hardness and wear resistance of the blade steel. Therefore, a comprehensive analysis is required to evaluate the cutting performance of knife blades. In this study, such an analysis was conducted based on a quantitative model to describe the cutting depth of paper cards containing SiO_2_ particles by steel blades, and major contributing factors were summarized. The effect of the micro-geometries of blade edges was thoroughly discussed, and a geometry factor ξ for the micro-geometry of a blade edge was introduced into the model. The experimental results indicated that mechanical processing could produce a rough blade edge and a higher ξ value, accordingly. A similar effect was caused by the carbides in the martensitic steels for blades, and the ξ value was found to increase linearly with the volumetric fraction of the carbides. The extraordinary cutting behavior of the 3V blade implied that fine coherent carbides may result in an efficient improvement (40–50%) in the total cutting depth.

## 1. Introduction

Metallic cutting tools have been utilized for over two thousand years since human civilizations prospered [1,2]. Though advanced techniques have emerged to cut food [3,4] or fabricate ceramics like KDP crystals [5,6,7] at this time, conventional cutting tools like knives still dominate in the kitchen and other domestic applications. People are continuously seeking to improve the cutting performance of knives and considering it as the most crucial property. To be specific, cutting performance usually means the capability to cut up materials and more importantly, the retention of the cutting up capability or sharpness. So far, techniques have been developed to evaluate the cutting performance of knives [8]. The cutting force can be measured to assess the sharpness in a knife-cutting process [9,10,11]. However, this simple criterion overlooks the complexity of the cutting process. Theoretically, cutting up is the fracture of materials triggered by elastic energy, which is stored in the material being cut by the blade edge. When the stored energy density surpasses a critical value that is related to the fracture toughness of the material, cracking starts to occur and results in the cutting up [12,13]. For a comprehensive description of cutting performance, the blade sharpness was quantified by a dimensionless Blade Sharpness Index (BSI) [14,15,16], which is the ratio of the energy required to initiate a cut over the product of cut depth, thickness and the fracture toughness of the testing material. The BSI parameter can effectively evaluate the instant sharpness of a blade. However, this technique is unable to reveal the evolution of the blade’s sharpness when the blade edge degrades during cutting. In the everyday use of steel blades, the degradation of a blade edge usually occurs due to abrasive wear [17] and/or a brittle fracture [18].

Fortunately, a standardized technique developed by the Cutlery and Allied Trades Research Association (CATRA) in Sheffield, England, has been introduced to evaluate both blade sharpness and its retention quantitatively [19,20]. According to the CATRA method (ISO8442-5:2004(E)) [21], the blade of a knife is designed to cut standard paper cards containing quartz abrasives for 60 cutting cycles. One cutting cycle means a to and fro movement of the knife blade, with respect to the paper cards. Since the blade edge turns from being sharp to blunt due to abrasive wear as the cutting proceeds, the instant sharpness and retention of sharpness can be defined by the total depth of the paper cards that have been cut in the initial 3 cycles and all 60 cycles, respectively. Up to date efforts have been made to study the improvement in the sharpness retention of steel blades via the CATRA technique, yet current studies only explore limited blade steels [20], and carbides have been found to play an important role in the cutting processes [22,23,24,25].

For the development of the blade industry, it is important and necessary to build up a comprehensive understanding of the cutting performance of steel blades, including their sharpness and its retention. Even though there are some pioneering studies on hair shaving by steel blades [18,26,27] and the corrosion resistance of steel blades [28], a more general application of blades in cutting bulk materials is still rarely discussed. As an pioneering exploration, a quantitative model [29] has recently been established between the cutting depth and wear loss of a blade. This model can guide the development of novel blade steel and quality control in industrial production; however, it is still in its infancy. The effects of influential factors in the model (e.g., the hardness of the steel, blade angle and width of the blade tip) still need to be assessed individually in a larger database of blade steels. Moreover, the polish finish of a blade edge has been found to affect the force applied in cutting meat [30], and a similar effect may also exist in the steel blades containing carbides, as the carbides can modify the profile of a blade edge in the cutting process [24]. Unfortunately, the effect of the micro-geometry of a blade edge was overlooked in the previous study [29].

In this study, the standard CATRA cutting tests were performed on the blades made of several martensitic steels. Both sharpness and its retention performances were evaluated and analyzed in terms of individual factors, like the blade angle and steel hardness. In particular, the micro-geometries of blade edges were intensively characterized, and their effect on cutting depth was evaluated quantitatively. Both the mechanical polish and carbides in steels can contribute to a rough blade edge, and a prospective steel for a robust, sharp blade was proposed.

## 2. Materials and Methods

### 2.1. Blade Steels

**Figure 1:**
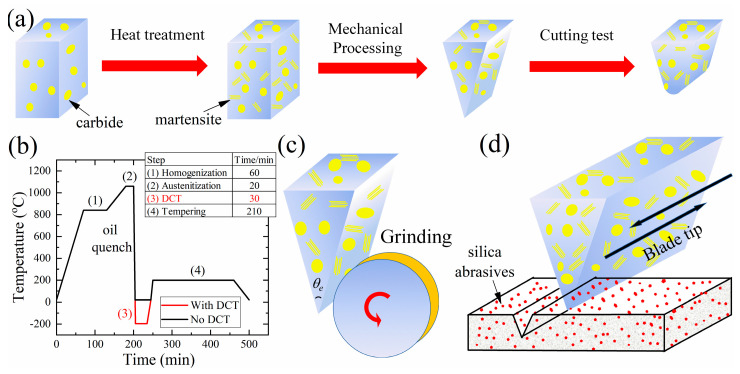
A study of the cutting performance of steel blades, following (**a**) the experimental procedures: (**b**) heat treatment with/without DCT and tempering on the raw steel plates, (**c**) mechanically fabricating the steel plates into a blade geometry with blade angle θ_e_ and (**d**) evaluating the cutting behavior of blades in the cutting tests.

The raw steel plates for knife fabrication were purchased commercially: MSSs (3Cr13, 5Cr15MoV, 7Cr17MoV and 9Cr18MoV) were purchased from Taiyuan Iron & Steel Co., Ltd. (Taiyuan, China), the powder steels 3V and S35VN from Crucible Industries LLC (New York, NY, USA) and M390 from Bohler-Uddeholm Corporation (Elgin, IL, USA). The chemical compositions of these steels were determined in the previous study [23], and the content of the major elements are listed in Table 1. Before mechanical processing, these steel plates were heat-treated following the procedures recommended by the producers (refer to the official data sheets), as shown in Figure 1b: firstly, they were homogenized at 840 °C and austenitized at a high temperature (1060~1150 °C), then martensitic transformation was triggered by the oil quench to improve the hardness and wear resistance. Tempering at lower temperatures (200 °C for MSSs, S35VN and M390; 540 °C for 3V and 4V) for 210 min was performed to relieve the internal stress caused by the martensitic transformation. To evaluate the effects of hardness and microstructure on the cutting depth, additional Deep Cryogenic Treatment (DCT) was performed on 9Cr18MoV, which contains the highest carbon content that stabilizes the austenite; therefore, the DCT would have the most significant effect in reducing the retained austenite. A series of tempering temperatures (170~250 °C) were applied to 5Cr15MoV, which contains a medium level of carbon.

### 2.2. Knife Blade Fabrication and Cutting Tests

The heat-treated steel plates were ground into a wedge shape, and the thinner ends were fabricated into blades using the emery wheel at the speed of 3000 rpm, as displayed in Figure 1c. The blade angle for each blade was measured using a laser goniometer. The cutting performance of these steel blades was evaluated, following the standard CATRA procedure [19,29] (ISO8442-5:2004E). The cutting behaviors of steel blades with various blade angles (18°~54°) were examined to evaluate the effect of blade geometry. The surfaces of the blades made of 5Cr15MoV were ground using a series of grinding papers (60#, 240#, 320# and 600#) to inspect how the blade surface finish affects the cutting performance. According to the American Standard (ANSI), the grinding papers were classified by the number of abrasive particles in a square inch; therefore, the grinding paper 600# with a median grit size of 15.3 μm produced a smoother sample surface than the 60# grinding paper, which contained much coarser SiC abrasives (median diameter 250 μm).

### 2.3. Materials Characterization

X-ray Diffraction (XRD) patterns were collected in the 2*θ* range of 40°~105°, at a step rate of 2°/min using a X’Pert PRO MPD X-ray diffractometer (Malvern PANalytical, Malvern, UK) equipped with a copper target (λ_CuKα_ = 0.15418 nm), and the diffraction peaks were detected to identify the carbide species at 40 kV and 40 mA. The microstructures of the blade steels were characterized using the scanning electron microscope (TESCAN CLARA, Brno, Czech Republic). The conventional Transmission Electron Microscopy (TEM) imaging was operated at 200 kV, and high-resolution elemental mappings were acquired using a Thermo Fisher Science Talos F200X (Thermo Fisher Science, Waltham, MA, USA). TEM specimens were prepared using a twin-jet electrochemical polisher (Smart Innovate, RL-2, Massachusetts Institute of Technology, Cambridge, MA, USA), operated at 18 V and −30 °C with a 5 vol.% perchloric acid electrolyte.

## 3. Results and Discussions

### 3.1. Microstructure and Phases of Blade Steels

**Figure 2:**
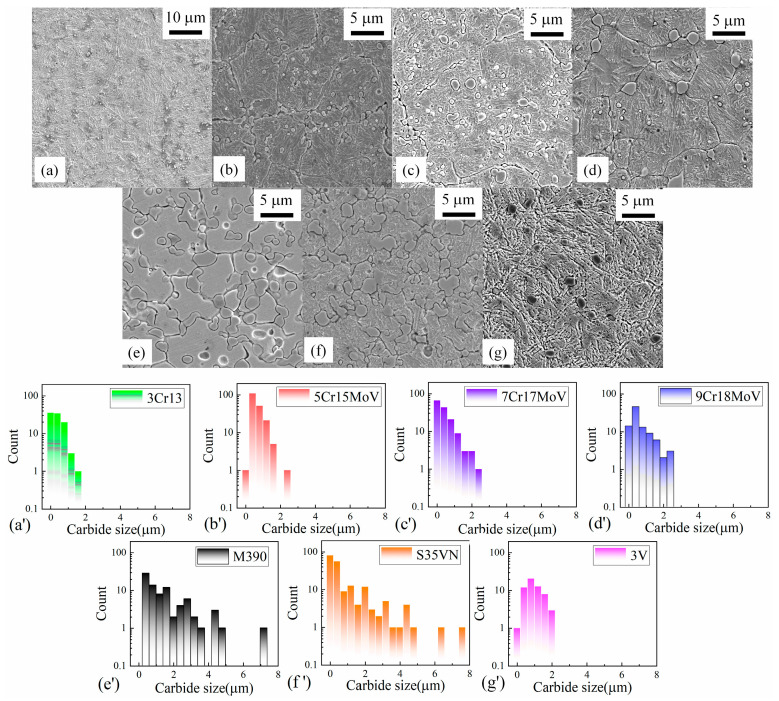
Microstructures of blade steels and size distribution of carbides imaged by SEM: (**a**,**a’**) for 3Cr13, (**b**,**b’**) for 5Cr15MoV, (**c**,**c’**) for 7Cr17MoV, (**d**,**d’**) for 9Cr18MoV, (**e**,**e’**) for M390, (**f**,**f’**) for S35VN and (**g**,**g’**) for 3V.

**Figure 3:**
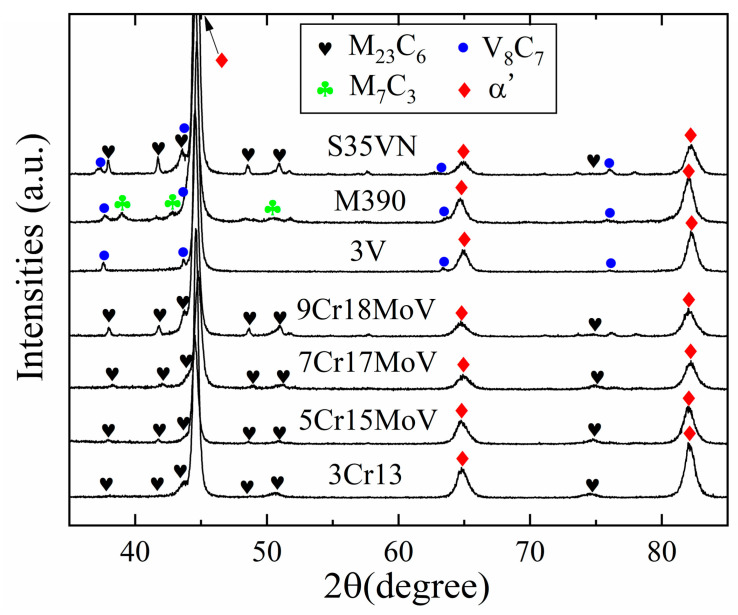
Carbide phases in the knife steels revealed by XRD.

**Figure 4:**
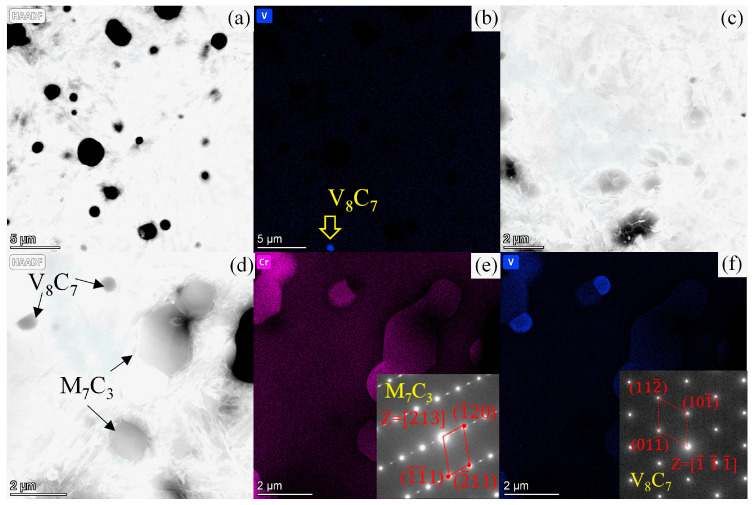
Microstructure of the steels: (**a**) HAADF-STEM image and mapping of element, (**b**) Vanadium for 3V, (**c**) HAADF-STEM image for 7Cr17MoV, (**d**) HAADF-STEM image and mapping of element, (**e**) Chromium and (**f**) Vanadium for M390, and carbides M_7_C_3_ and V_8_C_7_ were identified via the SAD patterns in the insets of (**e**) and (**f**), respectively.

The microstructures of the blade steels were inspected using the scanning electron microscope, as displayed in Figure 2a–g. Carbide particles and a martensitic matrix were observed in these SEM images, and the size distributions of the carbides are displayed in Figure 2a’–g’. It seems that the number and size distribution of the carbides increases with the carbon content in the steels. The types of carbides in these steels have been investigated using the Co-Kα emission, combined with elemental mapping analysis in a previous study [23]. Only M_23_C_6_ carbide was found in the MSSs, and there was more M_23_C_6_ carbide in the steels with a higher carbon content. The carbide particles were V_8_C_7_ in the powder steel 3V, whereas the dominating M_7_C_3_ and some minor vanadium carbides were found in M390. Both M_23_C_6_ and V_8_C_7_ existed in the steel S35VN. The effect of these carbides on the wear resistance and hardness were discussed in a previous study [23]. In this current study, the carbide phases were verified using X-ray diffraction with the Cu-Kα emission as displayed in Figure 3. The minor vanadium carbide in M390 was identified as V_8_C_7_, and the carbide V_8_C_7_ was confirmed by the Selected Area Diffraction (SAD) pattern using the Transmission Electron Microscopy (TEM) technique, as shown in Figure 4e,f.

### 3.2. Major Factors That Influence the Cutting Performance of Steel Blades

A previous study [29] has revealed that the blade edges of steel knives are prone to suffer abrasive wear during cutting, and this results in the deterioration of the cutting performance. A quantitative model has been established to describe the relationship between the sharpness retention and abrasive wear in steel blades. In this current study, the cutting performance of blades made of a broader range of martensitic steels were analyzed using this model. The influences of major factors like blade angle *θ_e_* and steel hardness on the cutting performance of blades were discussed individually.

#### 3.2.1. The Blade Angle *θ_e_*

**Figure 5:**
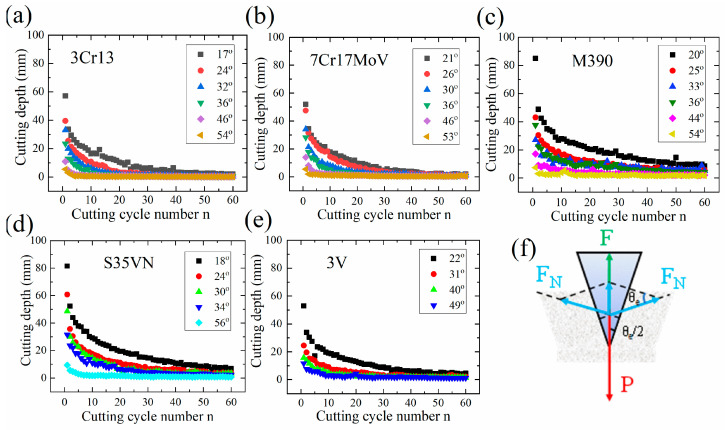
Cutting depth as function of the cutting cycle number n for the steel blades with various blade angles: (**a**) 3Cr13, (**b**) 7Cr17MoV, (**c**) M390, (**d**) S35VN and (**e**) 3V were achieved by a wedge-shape blade with a blade angle of *θ*_e_ in the diagram (**f**).

For the blades made of martensitic steels in Figure 5a–e, the cutting depth was found to be significantly influenced by the blade angle *θ_e_*. This remarkable effect probably arises from the strong dependence of the contact force F at the blade tip on the blade angle *θ_e_:* F = P − 2F_N_sin(*θ_e_*/2). A larger blade angle *θ_e_* lowers the contact force F and therefore brings down the contact pressure upon the paper cards by the blade edge; therefore, a blade with a larger blade angle *θ_e_* often cuts fewer paper cards per cycle. The contact pressure at the blade edge decreased continuously during cutting, due to the abrasive wear. This finally resulted in an early blunt-out of the blade, since a minimum pressure p_c_ (136 MPa [29]) was required to maintain cutting paper rather than grinding paper.

#### 3.2.2. Hardness of the Blade Steels

Hardness is another factor that affects the cutting performances of steel blades, because empirically the hardness of materials is approximately inversely proportional to the wear rate that closely correlates with the blade geometry. In practice, martensitic steels are excellent raw materials for knife blades due to their microstructures composed of a strong martensitic matrix and brittle, hard carbide particles. For a certain type of martensitic steel, its α’-matrix/carbide microstructure is evolving continuously during the heat treatment, and it exhibits a varying hardness accordingly, so it is essential to perform heat treatment properly.

**Figure 6:**
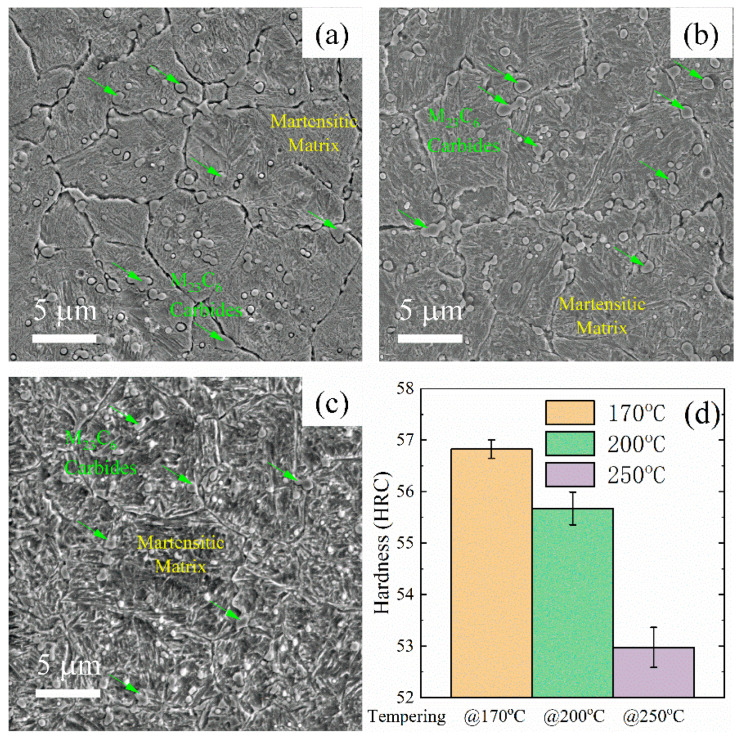
Microstructure of blade steel 5Cr15MoV imaged by SEM after tempering at (**a**) 170 °C, (**b**) 200 °C and (**c**) 250 °C for 2 h, respectively. The hardness of blade steel 5Cr15MoV decreased as the tempering temperature increased (**d**).

The hardness of steel 5Cr15MoV was found to decrease significantly with the increase of the tempering temperature, as shown in Figure 6d and Table 2. This softening in martensitic steels has been well accepted as a result of relieving processes during the tempering [31], e.g., carbon diffusion, the decomposition of martensite and a formation of ε-carbide, and this softening effect has been found in similar high-carbon martensitic steels previously [32,33]. However, these processes were on a sub-micro scale and are hardly discernible in the SEM images (Figure 6a–c).

**Figure 7:**
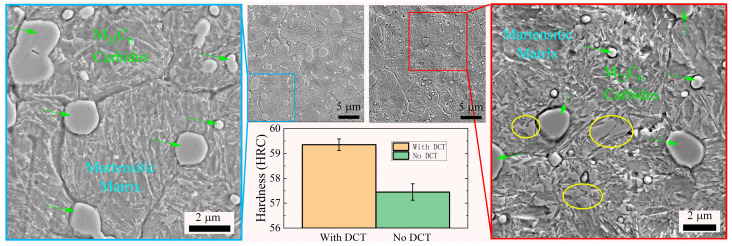
Comparison drawn between the microstructure of blade steel 9Cr18MoV imaged by SEM with DCT (**left**) and without DCT (**right**), and the higher martensitic transformation rate due to DCT raised the hardness of 9Cr18MoV.

The hardness of martensitic steel can be further enhanced after quenching via Deep Cryogenic Treatment (DCT). In the cryogenic treatment, the steel specimens were treated at −196 °C to boost the martensitic transformation process. The microstructures of steel 9Cr18MoV with and without DCT are compared in Figure 7. Highlighted by yellow circles, the areas surrounded by the needle-like martensite are residual austenite [34] that was unable to transform to martensite in the 9Cr18MoV after quenching without DCT, while these phases were rarely seen after DCT, and the matrix was filled with needle-like martensite. Consequently, the hardness was raised from HRC 57.45 ± 0.33 to HRC 59.35 ± 0.23 after DCT, as shown in Table 2.

**Figure 8:**
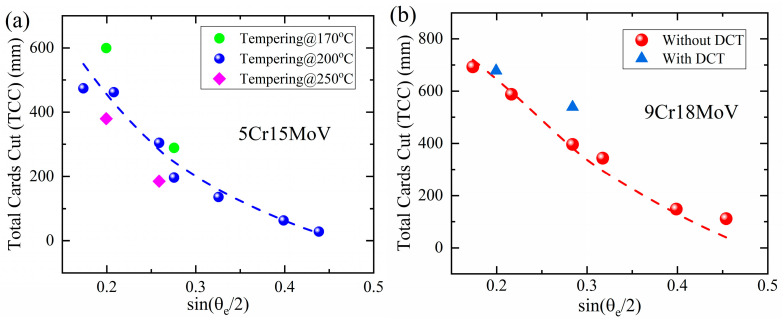
Correlation between cutting performances and hardness: (**a**) effect of tempering temperatures on the TCC by blades made of 5Cr15MoV; (**b**) effect of DCT on the TCC by blades made of 9Cr18MoV.

The total cutting depths of the blades made of tempered 5Cr15MoV and 9Cr18MoV with/without DCT were plotted as a function of sin(*θ_e_*/2) in Figure 8a,b, respectively. According to the model developed previously, the Total Cards Cut (TCC) can be written in the following form:

(1) $$\begin{array}{cc} TCC\left(H,\theta_{e}\right) & =\frac{M}{4d\tan\left(\frac{\theta_{e}}{2}\right)}\left(\frac{H}{K}\right)\left[w\left(60\right)-w\left(0\right)\right]\\ & \;\;=\frac{M}{4d}\sqrt{\frac{H}{K}\frac{8F}{\tan\left(\frac{\theta_{e}}{2}\right)}}(\sqrt{60+n_{0}}-\sqrt{n_{0}}) \end{array}$$

where *K* is the wear constant, *d* is the width of the paper cards that have been cut, M is the cutting depth coefficient of the steel blades and *w(n)* is the width of the blade edge as a function of cutting cycle number *n*. The total cutting depths by the blades made of 5Cr15MoV and 9Cr18MoV were found to largely obey Equation (1). Enhanced cutting depths were achieved by the harder blades made of 9Cr18MoV with DCT and 5Cr15MoV tempered at 170 °C; at the same time, the softer blade made of 5Cr15MoV tempered at 250 °C exhibited a smaller cutting depth.

**Figure 9:**
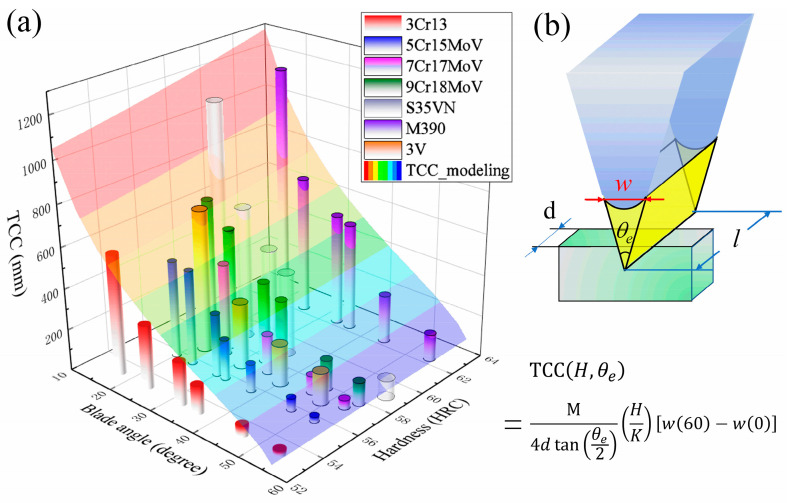
The cutting depth by steel blades are modeled in terms of blade angle *θ_e_* and the HRC hardness of the blade steels (**a**) using a quantitative model containing the width of blade edge *w* (**b**).

The total cutting depths by those blades made of various martensitic steels are summarized as a function of the blade angle *θ_e_* and HRC hardness in Figure 9, and the experimental data are modeled by the 2-dimensional surface of Equation (1), where the parameters M = 0.049, K = 9.56 × 10^−4^ and n_0_ = 3 for 9Cr18MoV [29] are adopted. For all the steel blades, a decreasing trend of the cutting depth with the increase of the blade angle was observed and is well described by Equation (1); on the other hand, the cutting depth does not follow a simple correlation with hardness, and a large discrepancy exists between the experimental data and the modeling with respect to the hardness of the steels. This disagreement may arise from the complexity of the abrasive wear process of the multiple-phase martensitic steels. Further analysis [23] on the martensitic steels with α’-matrix/carbide microstructures reveals the abrasive resistance can also be greatly improved by either small carbides strongly bonded to the martensitic matrix or a strengthened matrix.

#### 3.2.3. Merits and Limitations of the Quantitative Model

**Figure 10:**
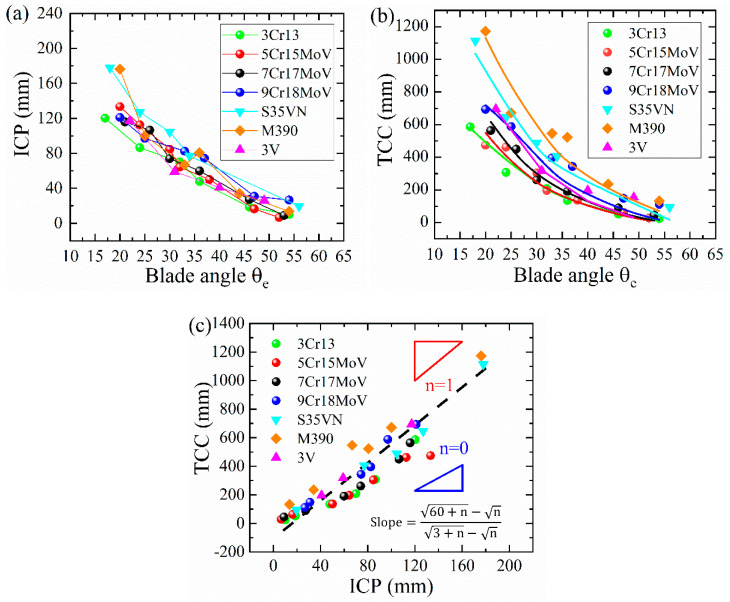
Dependence of (**a**) ICP and (**b**) TCC on the blade angle *θ_e_*, and (**c**) linear correlation between ICP and TCC was observed.

According to the ISO standard (ISO8442-5:2004E), the Initial Cutting Performances (ICPs) of steel blades were evaluated using the total depth of the paper cards that had been cut in the first 3 cutting cycles (Figure 10a), while the sharpness retention was revealed by the Total Cards Cut (TCC), thus demonstrating the total cutting depth in 60 cycles, as displayed in Figure 10b. A simple linear correlation was found between TCC and ICP for all steels, as shown in Figure 10c. This correlation can be quantified as $(\sqrt{60+n_{0}}-\sqrt{n_{0}}$)/($\sqrt{3+n_{0}}-\sqrt{n_{0}}$), since both TCC and ICP can be modeled as CCD(60) and CCD(3) based on the following quantitative relation:

(2) $$CCD(N)=\frac{M}{4d}\sqrt{\frac{8F}{\tan\left(\frac{\theta_{e}}{2}\right)}(\frac{H}{K})}(\sqrt{N+n_{0}}-\sqrt{n_{0}})$$

The abrasive resistance of these steels has been analyzed in the previous work [23], and the wear constant K for each steel was determined based on the volume loss of the blade edge, as listed in Table 3. The cutting behaviors of steel blades can be well modeled by Equation (1) in solid curves, as displayed in Figure 10b. It should be noted that a significant variation occurs in the M values for these steels in Table 3. In principle, M indicates the capability of the steel blade with a certain edge geometry to cut up paper cards, and the M value should only depend on the strength of the paper cards and the pressure achieved at the contact area between the blade edge and the paper cards. Since all the blades were fabricated into blade geometries with the same range of blade angle and the same paper cards were utilized in the cutting tests, therefore, a large variation in the M values probably implies the contact pressure at the blade edge may vary in a large range due to a dynamic contact status between the paper cards and the steel blade edge.

### 3.3. Technical Analysis on the Geometry of Blade Edge

#### 3.3.1. Width of Blade Edge *w*

**Figure 11:**
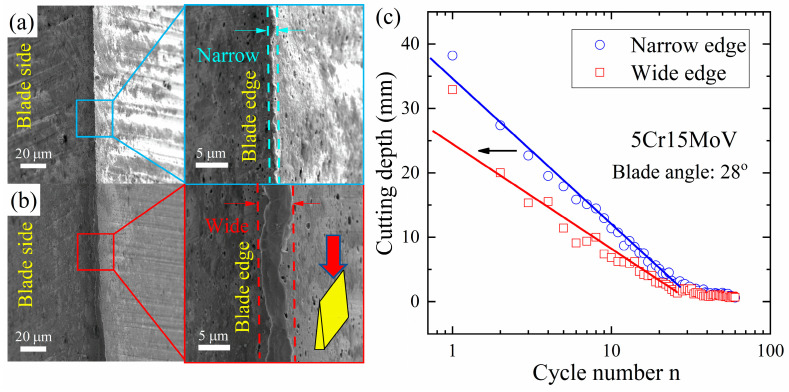
Comparison of top-down SEM images, as indicated by the red arrow, of 5Cr15MoV steel blade with a narrow edge (**a**) and a wide edge (**b**); and their cutting depths in paper cards (**c**).

The cutting capability of a blade is sensitive to the initial status of the blade geometry, even though the same blade angle has been fabricated. A comparison has been drawn between the total depth of paper cards cut by a narrow blade edge (Figure 11a) and a wide blade edge (Figure 11b) made of 5Cr15MoV in Figure 11c. Even if the blade angle of both these two blades was 28°, the narrow blade edge created higher contact pressure and resulted in a larger cutting depth. The initial status of the blade edge predominates the cutting performance over the entire cutting process.

#### 3.3.2. Micro-Geometries of Blade Edge

**Figure 12:**
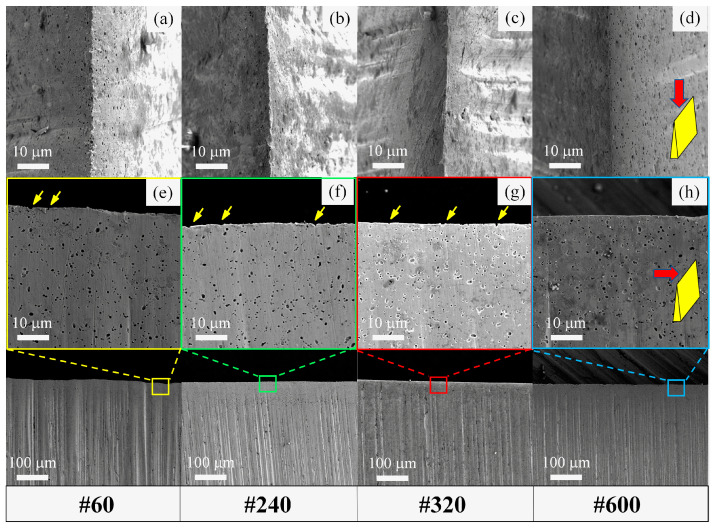
Top-down view (**a**–**d**) and side view (**e**–**h**) of SEM images of the blade edges made of 5Cr15MoV steel, which were ground into the wedge shape with the same blade angle of 27°using the grinding papers (**a**,**e**) 60#, (**b**,**f**) 240#, (**c**,**g**) 320# and (**d**,**h**) 600#.

Since the cutting behavior of a blade is highly sensitive to its initial condition, it is crucial to investigate the effect of the mechanical processing of a blade edge on cutting performance. This is because the mechanical grinding and polishing usually produces scratches on the processed surface and finally affects the micro-geometries of the blade edges, as these edges are actually intersections of two freshly processed surfaces [35]. In Figure 12a–d, it is found that the blade sides turned from rough to smooth when they were ground by finer sandpaper (from #60 to #600). Accordingly, the blade edges also become smoother from Figure 12e–h.

**Figure 13:**
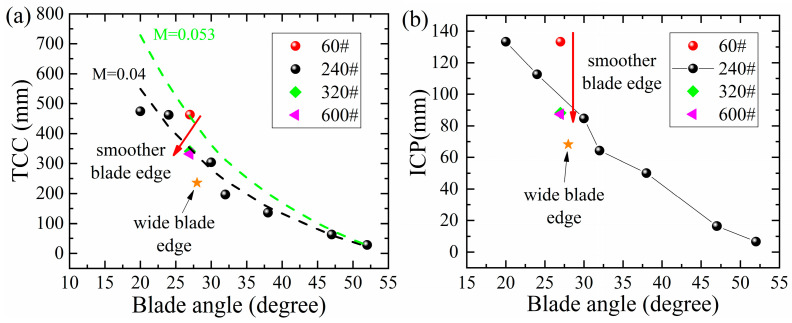
Cutting performance: (**a**) TCC and (**b**) ICP of blades made of 5Cr15MoV ground by different sandpapers.

The influence of the micro-geometry of a blade edge on cutting performance has been inspected and is presented in Figure 13. Obviously, a rougher blade edge resulted in a larger cutting depth, in both the TCC and ICP, whereas the cutting depth reached a limit when finer sandpaper (240# or higher) was used to grind the blade. That is because a rough blade edge has a reduced contact area with the paper cards and raises the effective pressure, therefore enhances the cutting effect. On the other hand, when ground by finer sandpaper, the contact area approaches a fixed value, and a stable cutting behavior is achieved. According to the modeling of Equation (2), a higher M value (from 0.04 to 0.053 mm/MPa) is determined for a rougher blade edge (from 240# to 60#), and the cutting depth can be enhanced by approximately 32.5%. It should be noted that the micro-geometries like burrs and scratches produced by mechanical processing are likely to be bent down [29,36] due to plasticity and finally worn away [29,36] by the continuous, abrasive wear during cutting. It would be interesting to conceive a mechanism to maintain the “sharpness” of the blade edge.

**Figure 14:**
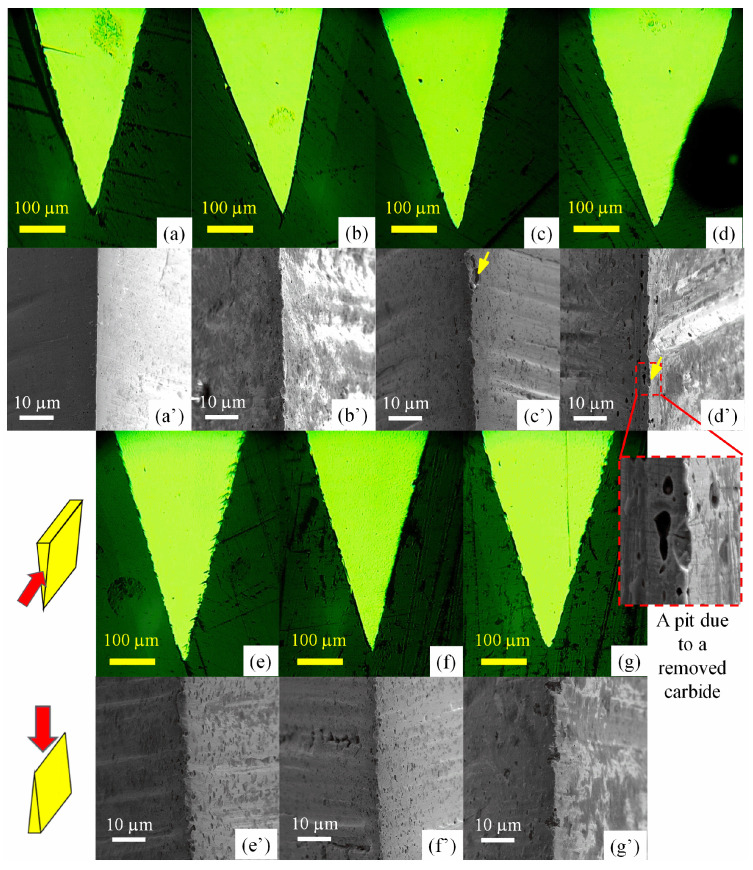
Cross section views of optical microscopic images (**a**–**g**) and top-down views of SEM images (**a’**–**g’**) reveal initial micro-geometries of blade edges made of various martensitic steels: (**a**,**a’**) 3Cr13, (**b**,**b’**) 5Cr15MoV, (**c**,**c’**) 7Cr17MoV, (**d**,**d’**) 9Cr18MoV, (**e**,**e’**) M390, (**f**,**f’**) S35VN and (**g**,**g’**) 3V. The red arrows indicate the direction to view the blade, and the yellow arrows mark the pits at the blade edges.

The large variation in the M values for martensitic steels in Table 3 can also be interpreted in terms of the micro-geometries of the blade edges, as shown in Figure 14. Small pits were observed at the edge, and more pits were found in the steel with higher carbon content, e.g., 9Cr18MoV. Electrographs have revealed that these pits may be formed after the pulling out of carbides, as marked by the yellow arrows in the top-down view of the blades magnified in Figure 14c’,d’ as well as in the side view of the blades in Figure 12f,g. In contrast, almost no pit is observed on the edge of 3Cr13 (Figure 14). Even though the carbides protruding from the steel matrix may also contribute to the blade edge roughness, a quantitative evaluation of the ratio of protruding carbides was unavailable due to a limitation of the characterization technique. Nonetheless, assuming a constant ratio of the pits/protruding carbides, it could still be found that the discontinuous features (pits and protruding carbides) of blade edges correlate with the varied M values obtained by modeling the cutting performance of steel blades previously. The blade made of carbide-free 3Cr13 exhibits the smoothest edge, and the M value for 3Cr13 is the lowest: 0.037 mm/MPa. For the martensitic stainless steels, the M value increases from 0.037 to 0.047 mm/MPa as the blade edges became rougher (from Figure 14a’–d’) due to an increasing of the carbide fraction in the MSSs. For those powder steels including 3V, S35VN and M390, the M values reach the highest level: 0.051~0.058 mm/MPa. The large variation of M values mainly arises from the rough tip edge naturally formed during mechanical fabrication as a result of the martensite/carbide microstructures of the steels. It is difficult to control the tip radius of the as-sharpened blade; instead, the blade width *w* (Figure 9) is favored to rationalize the cutting performance in the model (Equation (1)).

**Figure 15:**
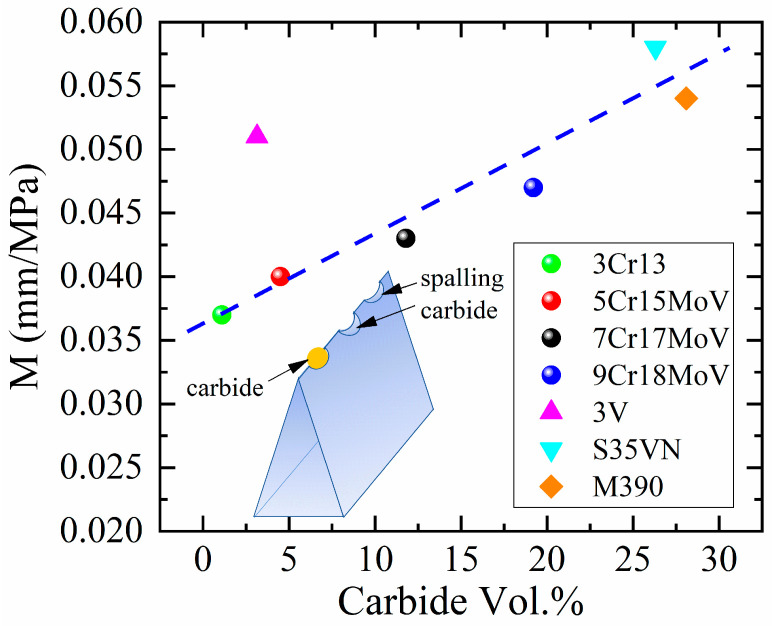
Linear correlation between the M values and carbide volumetric fraction in the martensitic steels.

The correlation between the M values and carbide fractions (Vol.%) in steels is revealed in Figure 15. It was not surprising to find the M value increased linearly with the amount of carbide for most martensitic steels, since more carbide usually causes more pits at the blade edges and thus rougher edge geometries and therefore sharper cutting behavior [29]. However, the M value for 3V obviously deviates from the linear relation. Considering its low carbide fraction, the M = 0.051 for 3V is unexpectedly high. There are, mainly, two possible causes of this phenomenon. Firstly, secondary hardening [37] occurs in the steel 3V when tempered at 540 °C, and carbon solutes diffuse out from martensite and form fine vanadium carbides [23], as displayed in the TEM images (Figure 4a,b). Most of the vanadium carbides dropped off after electrochemical treatment in the preparation of the TEM specimen, because 3V is more prone to suffer corrosion due to its lower Cr content than other steels. The remaining V_8_C_7_ particle is indicated by a white arrow in Figure 4b. In contrast, no such fine carbides were observed in MSSs, e.g., 7Cr17MoV in Figure 4c or in other powder steels, e.g., M390 in Figure 4d–f. Secondly, the fine V_8_C_7_ particles probably bonded strongly with a martensitic matrix because a coherent interface [38,39] could form between the vanadium carbides and α-Fe; furthermore, extraordinary wear resistance was also found in 3V, which may also have been contributed by the strong carbide cohesion. The excellent cutting behaviors of the 3V blade could be the synergetic effect of the two causes mentioned above. The fine vanadium carbides adhered firmly to the blade edge, while the martensitic matrix receded due to wear loss. Finally, micro-saws were produced at the blade edge, exhibiting a high M value and an outstanding cutting performance. To be noted, V_8_C_7_ particles also existed in M390, but these particles always have a larger neighboring carbide and therefore are less stable; moreover, much fewer V_8_C_7_ particles were found in M390 than in 3V due to the absence of secondary hardening, so no extraordinary cutting behavior beyond the linear modelling was observed in M390.

### 3.4. A General Quantitative Model

According to the previous discussion, it is necessary to introduce a factor ξ to describe the micro-geometry of a blade edge, and the cutting depth coefficient M is defined as M = M_0_ξ, where M_0_ = 0.037mm/MPa is the cutting efficiency factor of the ideal wedge-shaped blade, e.g., the blade made of 3Cr13. The typical range of ξ is determined to be 1~1.57, from the M values in Table 3. Subsequently, the quantitative model for cutting depth should be modified as follows:

(3) $$CCD(N)=\frac{M_{0}\xi }{4d}\sqrt{\frac{8FH}{K\tan\left(\frac{\theta_{e}}{2}\right)}}(\sqrt{N+n_{0}}-\sqrt{n_{0}})$$

**Figure 16:**
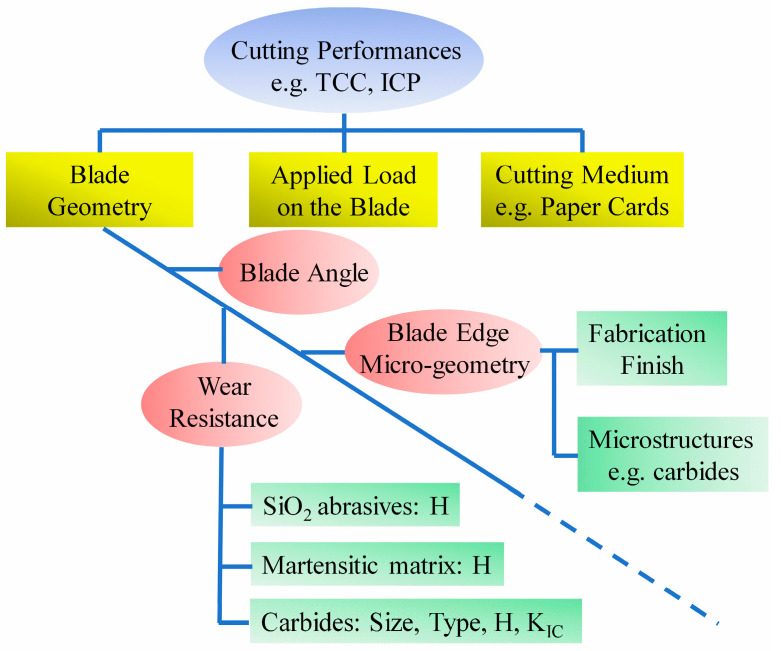
Summary of factors that influence the cutting behaviors of steel blades.

At the microscopic scale, cutting up is the fracture of materials caused by a blade, and the micro-geometry of a blade edge predetermines the actual force or contact pressure that the cutting medium is bearing. The factors like blade angle, wear resistance/hardness and blade micro-geometries discussed previously work together and determine the capability of a steel blade to maintain sufficient contact pressure upon the materials for cutting, as shown in Figure 16. Specifically, the contributions of these factors have been quantified in Equation (3). To retain the sharpness of a blade, the CCD can be enhanced by reducing K and $\theta_{e}$ and at the same time, increasing the hardness H and ξ. So far, significant achievements [23] have been made to increase the wear resistance of blade steels, and further improvement in this route has become very challenging. It is unrealistic to reduce $\theta_{e}$ excessively, because too small blade angle will impair the rigidity of the blade edge and may cause the edge to be turned easily. At the same time, further enhancement in the hardness of martensitic steels may cause the tipping of a blade edge due to a lack of toughness. For the improvement of cutting behavior, it is the most promising approach to introduce a robust, saw-like blade edge and thus a higher ξ, as it has the potential to raise the cutting depth by 40~50%, as shown in Figure 15. In practice, a rough blade edge can be fabricated by mechanical processing; however, the fabricated edge may be smoothened during cutting. An attractive idea is to introduce coherent hard particles (e.g., cubic-structured carbides NbC [40] and TiC [41]) into the blade steels. Such blades can self-sharpen during cutting, and no special mechanical process is required. In future, it would be appealing to explore this technique to produce blade steels containing fine coherent NbC or TiC particles.

## 4. Conclusions

In this study, the factors that influence the cutting performances of blades made of martensitic steels were thoroughly investigated, and the major findings are summarized below:

- A higher wear resistance/hardness and smaller blade angle were found to be favored to raise the cutting depth. The cutting performance can be adjusted by proper heat treatments (e.g., DCT and tempering);
- Sharpness was found to be sensitive to the micro-geometry of the blade edges, which may result from the grinding finish or the carbide/matrix microstructures of the steels;
- According to a quantitative analysis on the cutting data, the cutting depth coefficient M can be generalized to represent the roughness of a blade edge. The M values for almost all steels were found to follow a linear relation with the carbide volumetric fraction. More carbides result in a rougher blade edge;
- An exceptionally high M value of 0.051 mm/MPa was observed in 3V, which probably arises from a robust, rough blade edge due to the fine vanadium carbide being strongly bonded with the steel matrix;
- It is indicated that a rougher micro-geometry of a blade made of harder steel is the most favorable for a better cutting performance.

It is expected that these findings can facilitate the design of novel steels with excellent wear resistance and endow steel blades with the capability of self-sharpening.

## Figures and Tables

**Table 1:** Chemical composition of knife steels (wt%) [23].

Steels	C	Si	V	Cr	Mo	Others *	Fe
3Cr13	0.29	0.435	0.044	13.914	-	0.55	Bal.
5Cr15MoV	0.50	0.393	0.115	14.705	0.596	1.03	Bal.
7Cr17MoV	0.65	1.814	0.125	17.048	0.422	0.756	Bal.
9Cr18MoV	0.96	1.92	0.126	17.966	1.023	1.046	Bal.
3V	0.84	0.96	2.426	6.7	1.186	0.637	Bal.
S35VN	1.36	0.523	3.028	13.913	1.844	0.634	Bal.
M390	1.988	0.555	4.057	20.244	0.861	1.628	Bal.

* Other elements include Co, Ni, Mn and W; <0.5 wt% each.

**Table 2:** Hardness of steel 5Cr15MoV tempered at various temperatures and steel 9Cr18MoV with or without DCT.

Tempering Temperature (°C)	Hardness (HRC)	
	5Cr15MoV	9Cr18MoV
170	56.83 ± 0.18	/
200	55.68 ± 0.32	59.35 ± 0.23 (with DCT) 57.45 ± 0.33 (without DCT)
250	52.98 ± 0.39	/

**Table 3:** Modeling parameters for the cutting performances of steel blades.

Steels	HRC Hardness [23]	K (×10^−3^) [23]	Carbide Fraction (Vol.%) [23]	M (mm/MPa)
3Cr13	53.2	1.098	1.1	0.037
5Cr15MoV	55.7	1.18	4.5	0.04
7Cr17MoV	57.2	1.038	11.8	0.043
9Cr18MoV	57.8	0.956	19.2	0.047
3V	56.9	0.904	3.14	0.051
S35VN	58.9	0.955	26.3	0.058
M390	62.4	0.651	28.1	0.054

## Data Availability

The data presented in this study are available on request from the corresponding author.
